# Functional Changes Induced by Orexin A and Adiponectin on the Sympathetic/Parasympathetic Balance

**DOI:** 10.3389/fphys.2018.00259

**Published:** 2018-03-22

**Authors:** Antonietta Messina, Marcellino Monda, Anna Valenzano, Giovanni Messina, Ines Villano, Fiorenzo Moscatelli, Giuseppe Cibelli, Gabriella Marsala, Rita Polito, Maria Ruberto, Marco Carotenuto, Vincenzo Monda, Andrea Viggiano, Aurora Daniele, Ersilia Nigro

**Affiliations:** ^1^Department of Experimental Medicine, University of Campania “Luigi Vanvitelli,” Naples, Italy; ^2^Department of Clinical and Experimental Medicine, University of Foggia, Foggia, Italy; ^3^Struttura Complessa di Farmacia, Azienda Ospedaliero-Universitaria, Foggia, Italy; ^4^CEINGE-Biotecnologie Avanzate Scarl, Naples, Italy; ^5^Department of Mental Health, Physical and Preventive Medicine, Clinic of Child and Adolescent Neuropsychiatry, Università degli Studi della Campania “Luigi Vanvitelli,” Naples, Italy; ^6^Clinic of Child and Adolescent Neuropsychiatry, Center for Childhood Headache, Department of Mental Health, Physical and Preventive Medicine, University of Campania “Luigi Vanvitelli,” Naples, Italy; ^7^Department of Medicine and Surgery, University of Salerno, Baronissi, Italy

**Keywords:** orexin A, adiponectin, ICV-injection, heart rate, body temperature

## Abstract

Obesity and lifestyle-related diseases are major problems faced by people in developed nations. Although exercise training prevents the progression of diabetes and obesity, the motivation for exercise is generally low in obese animals and humans. The autonomic nervous system (SNA) plays a crucial role in the regulation of eating behavior. Moreover, the SNA is involved in the body temperature regulation that is strictly related to body weight control, in accordance with the “thermoregulatory hypothesis” of food intake. Some neuronal peptides and hormones, like orexins and adiponectin, are also involved in the regulation of locomotion activity as well as food intake and metabolic rate. Furthermore, adiponectin as well as orexin A are involved in the control of body temperature, food intake and therefore in obesity-related diseases. The aim of this study was to investigate the changes in body temperature (Tc), and heart rate (HR) after an intracerebroventricular (ICV) injection of orexin A and adiponectin in animal model. The results of this study show that the orexin A levels are likely involved in the increase of Tc and HR. It is also clear that there is not a correlation between these parameters and adiponectin levels. Further studies are needed to assess adiponectin actions and outcome in the central nervous system in terms of energy expenditure, body temperature, heart rate and physical activity performance regulation.

## Introduction

Obesity and lifestyle-related diseases are major problems faced by people in developed nations. Although exercise training prevents the progression of diabetes and obesity (Miyatake et al., 2015; Accetta et al., 2016), the motivation for exercise is generally low in obese animals and humans. Increasing motivation for exercise is the best treatment for obesity. In fact, the education on the importance of exercise training improves motivation level for exercise (Phillips et al., 2004; Sessa et al., 2011; Willems et al., 2017). Some neuronal peptides and hormones, like orexins and adiponectin, are also involved in the regulation of locomotion activity as well as food intake and metabolic rate (Miyatake et al., 2015).

As well described in the literature, the autonomic nervous system (SNA) plays a crucial role in the regulation of eating behavior. Moreover, the SNA is involved in the body temperature regulation that is strictly related to body weight control, in accordance with the “thermoregulatory hypothesis” of food intake (Messina et al., 2013).

The hypothalamic neuropeptide “orexin A/hypocretin 1” (de Lecea et al., 1998) causes a widespread stimulation of the sympathetic nervous system. This peptide was so named for its effects also on eating behavior (Ohno and Sakurai, 2008; Messina et al., 2014). However, an intracerebroventricular (ICV) administration of orexin A also causes tachycardia (Monda et al., 2005; Bertozzi et al., 2017), associated with an increase in blood pressure (BP) (Shirasaka et al., 1999; Avola et al., 2004) and metabolic rate (Lubkin and Stricker-Krongrad, 1998). These autonomic changes suggest that this peptide is involved in the regulation of autonomic reactions. In the light of these reports, this neuropeptide influences the food intake, inducing body temperature adaptations (Bafunno et al., 2014; Salomone et al., 2014; Messina et al., 2016). Indeed, an ICV-injection of orexin A causes an enhancement of the sympathetic discharge to interscapular brown adipose tissue (IBAT) in rats, and this sympathetic activation is associated with an increase in IBAT and colonic temperatures (Tc) (Kuru et al., 2000; Bayer et al., 2001; Monda et al., 2001; Mieda et al., 2004; Neri et al., 2009b; Messina et al., 2015). The tachycardia and hyperthermia caused by an ICV administration of orexin A suggest that this neuropeptide causes a widespread stimulation of the sympathetic nervous system.

The complexity of the “obesity problem” has become clearer since adipose tissues have been recognized as an endocrine organ that produces biologically active substances defined as “adipokines,” protein hormones with pleiotropic functions in the regulation of energy metabolism insulin sensitivity, inflammation, atherosclerosis, and proliferation. Adiponectin, which is mainly produced in white adipose tissue (WAT), characteristically differs from most adipokines since it is negatively correlated with obesity. Adiponectin exerts pleiotropic beneficial effects mediated by the specific receptors AdipoR1, AdipoR2, and T-cadherin (Hug et al., 2004; Forlano et al., 2011; Nigro et al., 2014). The physiological role of adiponectin is related to its ability to reduce glucose, triglycerides, and free fatty acids playing a major role in the pathogenesis of metabolic syndrome (Shehzad et al., 2012; Petito et al., 2016). In addition, numerous experimental and clinical observations have shown decreased adiponectin bioactivity in obesity and obesity-related complications, including insulin resistance, diabetes, cardiovascular diseases, and non-alcoholic fatty liver disease (Shehzad et al., 2012). Recently, an important role of adiponectin was evidenced in vascular functions inflammation and immunity (Niinaga et al., 2016; Pecoraro et al., 2017).

Adiponectin as well as orexin A are involved in the control of body temperature, food intake and therefore in obesity-related diseases. Thus, the aim of this study was to investigate the changes in body temperature (Tc), and heart rate (HR) after an intracerebroventricular (ICV) injection of orexin A and adiponectin in rats.

## Materials and methods

### Animals

Male Sprague-Dawley rats (*n* = 24, divided into 3 groups of 8 animals each), 3 months old and weighing 250–300 g were used in the experiments. The rats were housed in pairs at controlled temperature (22 ± 1°C) and humidity (70%) with a 12:12 h light–dark cycle with the light on from 07:00 to 19:00 h. All experiments were carried out in accordance with the National Institute of Health Guide for the Care and Use of Laboratory Animals, revised 1996 or the UK Animals (Scientific Procedures) Act 1986 and associated guidelines, or the European Communities Council Directive of November 24, 1986 (86/609/EEC). All protocols respected the guidelines for investigation of experimental pain inconscious animals. The number of animals involved were kept to the minimum necessary for the purposes of the experiments. All these parameters were unaffected by the treatment comparing to untreated rats. The present study received the appropriate approval by the ethical committee of the University of Salerno.

### Apparatus

Thermocouples (Ellab) were used to monitor colonic temperature (Tc) and the values were stored on a chart recorder. Two electrodes applied to the forelegs monitored the heart rate (beats/min). Electrical signals were addressed to a polygraph (Dynograph, Beckman) to record the electrocardiographic activity on the card and on a computer disk.

### Drugs and doses

We used orexin A commercialized by Sigma–Aldrich (Italy) and home-produced adiponectin (Nigro et al., 2016). A dose of 0.1 μg/g of adiponectin and of 1.5 nmol of orexin A (dissolved in 5 μl of 0.9% NaCl sterile solution) was utilized for ICV-injection. 1.5 nmol of orexin A is a sub-maximal dose in the induction of hyperthermia, as demonstrated in previous experiments (Monda et al., 2003a).

### Procedure

All animals were anesthetized with ip pentobarbital (50 mg/kg bw) and a 20-gauge stainless guide cannula was positioned stereotaxically above a lateral cerebral ventricle at the following coordinates: 1.7 mm lateral to the midline, 0.4 mm posterior to the bregma, 3.0 mm from the cranial theca. The rats were given 7–10 days to recover from surgery judged by the recovery of preoperative body weight.

After recovery, the animals were anesthetized with ethyl urethane (1.2 g/kg bwip) and mounted in a stereotaxic instrument (Stoelting). The level of anesthesia was kept constant and evaluated by skeletal muscle relaxation, eye and palpebral responses to stimuli.

The heart rate and Tc were monitored at the same time. Tc was measured by inserting the thermocouple into the colon 4 cm from the anus. These variables were recorded before (Time 0) of the ICV-injection of orexin A (1.5 nmol, group 1), or adiponectin (0.1 μg/g group 2) or saline (5 μl of 0.9% NaCl sterile solution group 3) into the lateral cerebral ventricle and over a period of 180 min after the ICV-injection. The ICV-injections were delivered into the left cerebral ventricle by gravity over 2 min. The injected volume was well controlled using a transparent polyethylene tube with a graduation of microliters. The cannula for the injection was 0.4 mm longer than the guide cannula. At the end of the experiment, the rats were then injected with an overdose of pentobarbital (200 mg/kg bw).

### Statistical analysis

Data were analyzed using the GraphPad Prism 6 software for Windows (Microsoft, USA). The analysis of variance for repeated measures (ANOVA) was used to determine differences among the variables after ICV-injection of Orexin A and adiponectin. When indicated by a significant *F*-value, a *post-hoc* test using the Tukey multiple comparisons was performed to identify significant differences between times. All data were reported as means ± SD. Statistical significance was considered for *p* ≤ 0.05.

## Results

**Figure 1:**
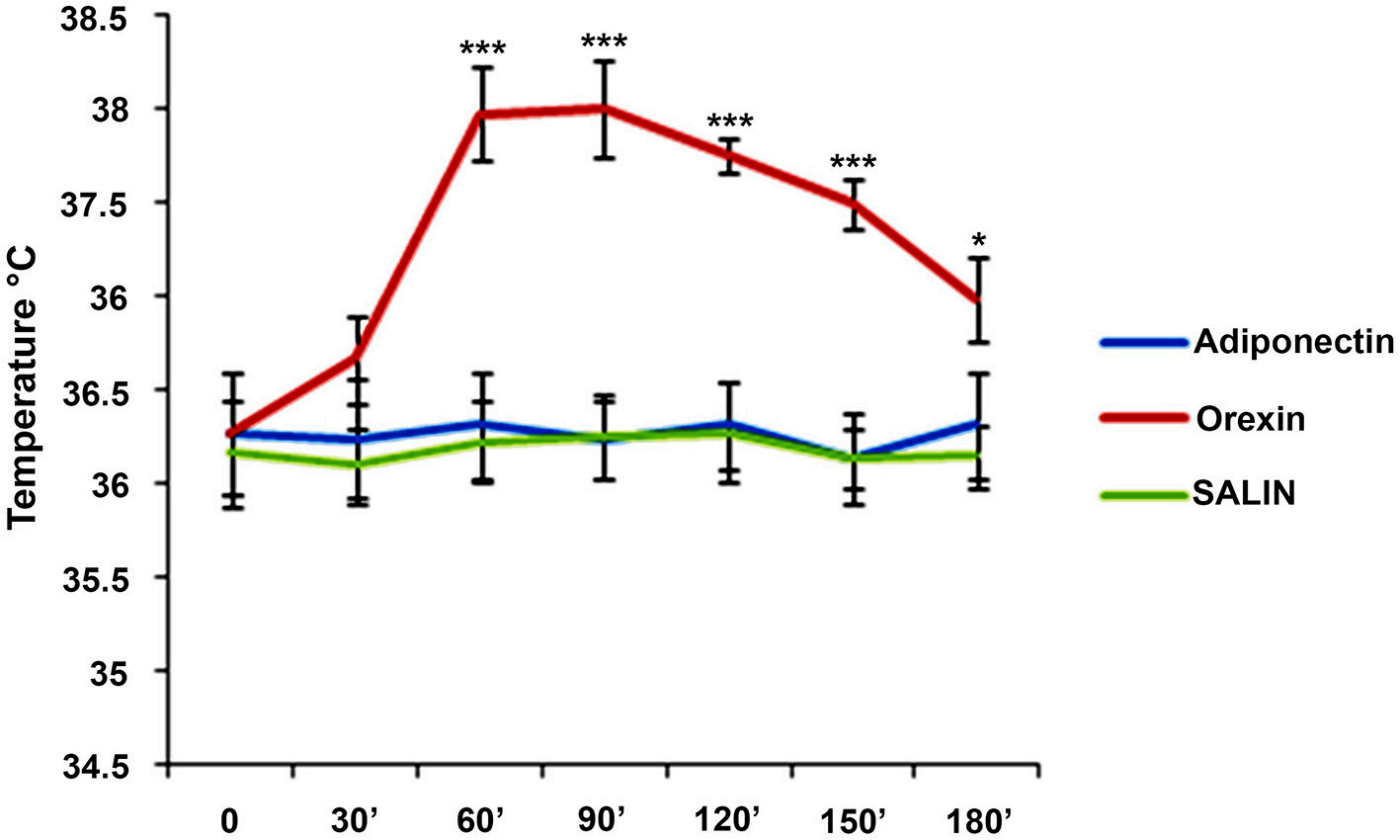
Means ± SD of values of T_c_. Intracerebroventricular (icv) injection of adiponectin (0.1 μg/g), or saline or orexin A (1.5 nmol) was made at time 0 and they were monitored each 30 for 180 min. post-injections. ^***^p < 0.001; ^*^p < 0.05.

A single dose of 1.5 nmol orexin A, 0.1 μg/g adiponectin, and saline solution were administered respectively to group 1, group 2, and group 3. For each group, T_c_ was monitored before of the ICV-injection (Time 0) and at the time of 30, 60, 90, 120, 150, 180 min after ICV-injection. ICV-injection of adiponectin and saline solutions, did not induced changes in T_c_ (*F* = 0.43; *p* > 0.05) (*F* = 0.69; *p* > 0.05), while a significant increase emerged after ICV-injection of the orexin A (*F* = 63.52; *p* < 0.001) (Figure 1).

**Figure 2:**
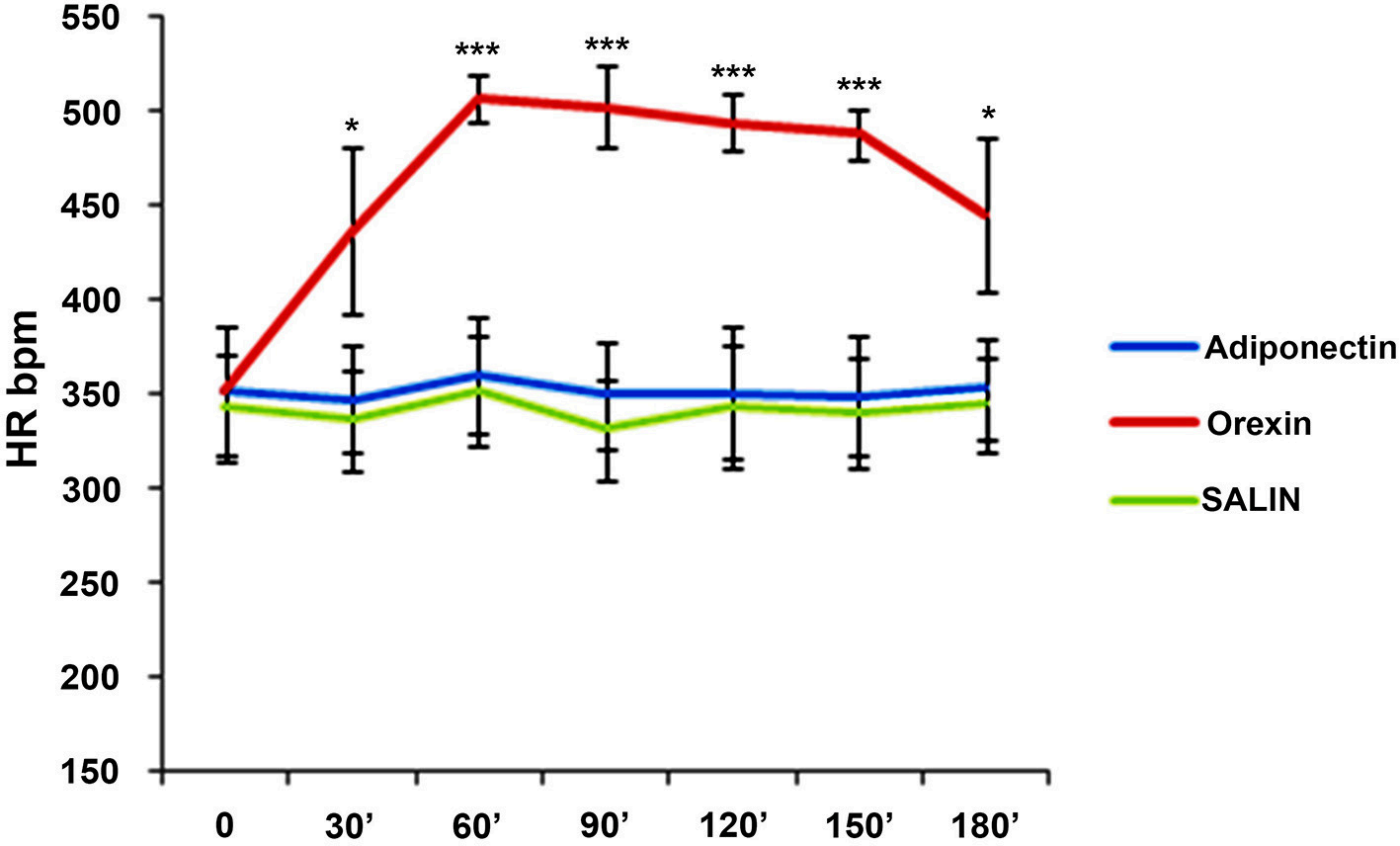
Means ± SD of values of HR. Intracerebroventricular (icv) injection of adiponectin (0.1 μg/g), or saline or orexin A (1.5 nmol) was made at time 0 and they were monitored each 30 for 180 min. post-injections. ^***^p < 0.001; ^*^p < 0.05.

In addition, after the ICV-injection of adiponectin and saline solution, there were no changes in heart rate (*F* = 0.16; *p* > 0.05) (*F* = 0.48; *p* > 0.05) while a statistically increase emerged after the orexin A ICV-injection (*F* = 26.41; *p* < 0.001) (Figure 2).

## Discussion

The main finding of the present study is that an ICV-injection of orexin A induces an increase in Tc and heart rate, while an ICV-injection of adiponectin or saline solution does not induce any change in the same parameters.

In particular, our results showed that from 60 to 180 min after the ICV-injection of orexin A there was a statistically increase in body temperature. Furthermore, our results showed that from 30 to 180 min after the ICV-injection of orexin A there was a statistically increase in heart rate (HR). The increase in Tc after ICV-injection of orexin A emphasizes the effect of this hormone on “core” temperature and suggest it among the peptides controlling body temperature (Monda et al., 2003b; Cerame et al., 2008; Cappellani et al., 2013).

In a recent study (Gubin et al., 2017), the high Tc level is related to type 2 diabetes mellitus (T2DM). In fact, in subjects suffering from T2DM, the thermoregulation is impaired, particularly the capacity to dissipate heat is reduced (Cavallaro et al., 2011; Kenny et al., 2016). Gublin et al. also described that thermoregulation is compromised already in the prediabetic (PD) state. Furthermore, a correlation between high level of HR and PD/T2DM state was described.

At the light of the results described in the present study, the orexin A could play an important role in the evolution of the PD/T2DM state. These findings support the hypothesis that the orexin A could be considered as an emerging biomarker for various endocrine disorders including diabetes mellitus and obesity which ultimately leads to various cardiovascular risk factors (Monda et al., 2006; Di Rosa et al., 2013; Bramanti et al., 2016; Mondola et al., 2016; Rani et al., 2017).

Different studies regarding adiponectin role on glucose homeostasis showed that this adipocytokine increases the insulin sensitivity in peripheral tissues; on the other hand, decreased plasma adiponectin levels in obesity and type 2 diabetes contribute to insulin resistance (Wei et al., 2017). Therefore, adiponectin is widely known as an anti-diabetic adipocytokine (Kadowaki et al., 2006; Neri et al., 2009a, 2013; Cianci et al., 2016). In this study, however, we observed that an ICV-injection of adiponectin in rats did not modify Tc and heart rate. Previously, Qi et al. showed that an intracerebroventricular administration of adiponectin decreases body weight mainly by stimulating energy expenditure but not through the inhibition of food intake (Qi et al., 2004; Giallongo et al., 2011). Furthermore, regarding exercise, a previous study conducted in rats have shown that an ICV-injection of adiponectin decreases locomotion activity in the home cage especially in the active phase without significant changes in food intake and oxygen consumption (Miyatake et al., 2015). Finally, Van De Wielle and Michels (2017) performed an interesting study analyzing a longitudinal association of Adiponectin with HRV in Belgian Children. They described that low adiponectin levels are unfavorable for the autonomic balance as measured with HRV and consequently for the cardiovascular risk, even during childhood; these findings was independent of body fat%. The results of our study are in line with these previous results that demonstrated how adiponectin reduces activity thermogenesis induced by physical and possibly even non-exercise activity via the central nervous system (Bramanti et al., 2012; Miyatake et al., 2015). Moreover, it was also observed that an injection of adiponectin into the lateral ventricle in sports rats did not decrease wheel-running activity (Morishima-Yamato et al., 2005).

In conclusion, this study indicates that an ICV of adiponectin in rats has different results on Tc and heart rate if compared to orexin A. The orexin A levels are likely involved in the increase of Tc and HR. It is also clear that there is not a correlation between these parameters and adiponectin levels.

Further studies are needed to assess adiponectin actions and outcome in the central nervous system in terms of energy expenditure, body temperature, heart rate and physical activity performance regulation.

## Author contributions

AM, IV, RP, and MR: carried out biological assays and, with the contribution of AV, GM, and GiM carried out the participants evaluations; AM, GC, MM, MC, AVi, AD: participated in the design of the study; FM and EN: performed the statistical analysis; AM and EN: conceived of the study, participated in its design, and coordination, and helped to draft the final manuscript. All authors read and approved the final manuscript.

### Conflict of interest statement

The authors declare that the research was conducted in the absence of any commercial or financial relationships that could be construed as a potential conflict of interest. The reviewer EM declared a shared affiliation, with no collaboration, with several of the authors, to the handling Editor.
